# Examining knowledge and traditional practices of mothers with children under five in Turkey on diarrhoea according to education levels

**DOI:** 10.1080/07853890.2022.2044508

**Published:** 2022-02-28

**Authors:** Cevriye Yüksel Kaçan, Aylin Palloş, Güven Özkaya

**Affiliations:** a Department of Public Health Nursing, Faculty of Health Sciences, Bursa Uludağ University, Bursa, Turkey; b Department of Fundamentals of Nursing, Faculty of Health Sciences, Bursa Uludağ University, Bursa, Turkey; c Department of Biostatistics, Faculty of Medicine, Bursa Uludağ University, Bursa, Turkey

**Keywords:** Mothers, child, knowledge, traditional practices, diarrhoea

## Abstract

**Background:**

Diarrhoea still ranks among the top causes of the deaths of children under five years old in the world. In solving this important health problem, it is necessary and imperative to know the health-related knowledge levels of mothers who take care of the child individually and the traditional practices they perform when their children are sick, in order to provide effective health education.

**Aim:**

This study aims to examine the knowledge levels and traditional practices of mothers with children younger than 5** **years old regarding diarrhoea in relation to their education levels.

**Methods:**

We conducted a cross-sectional web-based survey. The population of this cross-sectional study consisted of mothers with children under the age of 5 who lived in the metropolitan city Bursa in the South Marmara Region of Turkey. The survey was applied among the mothers of children under the age of 5 using the snowball sampling method *via* mobile platforms. The data were collected *via* Google Forms using a “Socio-Demographic Data Collection Form”, an “Information Form on Measuring the Knowledge Level of Mothers on Diarrhoea” and a “Form on Main Traditional Practices Used When Children Have Diarrhoea in Turkey” prepared by the researchers after a review of the relevant literature.

**Results:**

In the study, the mean total diarrhoea knowledge score of the participating mothers was found to be 22.01** **±** **3.72 (high). Multiple linear regression analysis was used to determine the relationship between the total diarrhoea knowledge scores of the participants and other variables. The difference in the knowledge scores based on education levels was statistically significant (*p*** **<** **.001). The most prevalently preferred traditional practice in the case of children's diarrhoea was “feeding the child banana” (92.5%).

**Conclusion:**

Maternal education level is determined to be a significant variable that positively affects diarrhoea knowledge levels.

**KEY MESSAGES**
- Diarrhoea continues to be among the top five preventable causes of death in the world and Turkey among children under the age of 5.
- The knowledge level of mothers about diarrhoea plays an important role in diarrhoea management. The level of knowledge about diarrhoea differs according to the education level of mothers.
- Traditional practices have an important place in the management of diarrhoea by mothers.

## 1. Introduction

Diarrhoea is a serious health issue in places where environmental conditions adversely affect health, there is no food sanitation, and individuals have insufficient knowledge about basic hygiene. Although the frequency of defaecating per day varies from person to person and according to diet, diarrhoea is defined as having more watery stools than normal and defaecating more often than 3 times a day [1,2]. According to the World Health Organization data, the total annual number of mortalities under the age of 5 worldwide dropped from 12.6 million in 1990 to 5.2 million in 2019 [3]. However, when we examine the causes of child mortality, diarrhoea continues to be among the top five preventable causes of death in the world and Turkey [3,4].

In Turkey, where the mortality rate under the age of 5 is 11.3 per thousand, pneumonia and diarrhoea are the leading causes of death. The prevalence of diarrhoea in this age group is 23% [4]. In previous studies on the topic, it may be seen that mothers' misinformation, attitudes and practices regarding diarrhoea are among the most influential causes of child deaths due to diarrhoea in Turkey and other countries [5,6]. According to the information in the literature, especially in developing and underdeveloped countries, the traditional practices of mothers to relieve diarrhoea have an important place, and diarrhoea is treated with traditional methods without any medical treatment [7,8]. Although traditional beliefs and practices passed down from the previous generation and passed on to the next generation vary from region to region, family to family and person to person, they still maintain their vitality and influence [5]. While solving an important health issue such as diarrhoea, which is one of the leading causes of child mortality, it is of great importance to learn about the knowledge levels of mothers, who take care of their children in person, about diarrhoea, as well as their traditional practices [9,10]. This is because improper traditional practices for the prevention and treatment of diarrhoea under the age of 5, when growth is fast, and sensitivity to external factors is high, aggravates the prognosis of diarrhoea in children and may even cause infant or child deaths [5,11,12].

Children are affected more severely by adverse environmental conditions, they cannot overcome problems such as many diseases and nutritional deficiencies on their own, and they require special help and attention from their environment [13]. Mothers' knowledge and practices regarding diarrhoea symptoms, the prevention and control of diarrhoea and diarrhoea management play an important role in reducing the rates of morbidity and mortality associated with diarrhoea in children under five years of age. Therefore, evaluating the knowledge levels and practices of mothers will help determine health education strategies to empower them. The number of studies on this topic in Turkey is low, and there is no study examining the effects of mothers' education levels on their traditional practices and knowledge levels about diarrhoea. Therefore, this study was planned to examine the knowledge levels and traditional practices of mothers with children under the age of 5 on diarrhoea based on their education levels.

## 2. Materials and methods

### 2.1. Study design and population

We conducted a cross-sectional web-based survey. The population of this cross-sectional study consisted of mothers with children under the age of 5 who lived in the metropolitan city of Bursa in the South Marmara Region of Turkey between 30.08.2020 and 30.10.2020. The survey was implemented among the mothers of children under the age of 5 using the snowball sampling method *via* mobile platforms. Owing to the ongoing COVID-19 pandemic, the mothers could not be safely contacted in person, and the link of the web forms was shared with the mothers. With data from the pilot study that we conducted with 30 mothers, the effect size was determined as 0.20 for the total Diarrhoea Knowledge Score (DKS). Thus, the power calculation for the present study was based on an effect size of 0.20, a standard deviation of 4.2 and an alpha level set at 0.05. The sample size required to obtain a power of 0.8 under these assumptions was 70 mothers for each education level (primary school, high school, bachelor's degree and postgraduate degree). To compensate for potential data losses due to incorrectly or incompletely filled forms, the forms were administered to more mothers than the required sample size (*n*** **=** **577).

### 2.2. Data collection tools

The data were collected online using the Google Forms platform. The first part of the form started with an informative text which stated information on the study and that participation in the study was on a voluntary basis, the collected data would only be used for the purpose of the study, and the data would not be shared with other persons/institutions. The form had a “yes/no” button that questioned the consent of the potential participants to participate in the study after receiving information. The mothers who marked the option “yes” and provided consent could move on to the survey and answer the questions. The second part was a “Socio-Demographic Data Collection Form” (17 items), the third part was an “Information Form on Measuring the Knowledge Level of Mothers on Diarrhoea” (26 items), and the fourth part was a “Form on the Main Traditional Practices Used When Children Have Diarrhoea in Turkey” (25 items). It took each participant an average of 10** **min to answer the forms.

#### 2.2.1. Socio-demographic data collection form

The form prepared by the researchers after the literature review [7,10,14–16] consisted of 16 questions such as the mother's age, her age of marriage, age at first childbirth, total number of children, number of children under 5** **years old, age of the child under 5** **years old, education level, marital status, employment status, family income, family type and occurrence of diarrhoea at least once in the child's life.

#### 2.2.2. Information form on measuring the knowledge level of mothers on diarrhoea

The form prepared by the researcher after the literature review consisted of 26 items for collecting information on the diarrhoea-related knowledge levels of the mothers [7,10,17,18]. The participants were asked to select the appropriate option among “true”, “false” and “I don't know” for each item. The content validity of the scale was analysed by calculating the content validity index (CVI). In order to evaluate the first version of the form consisting of 33 items, a total of 10 experts in the fields of Public Health (4 experts) and Paediatrics (6 experts) were consulted. The experts were asked to rate the scale items in terms of clarity and their relevance to the study, on a 4-point ordinal scale (1: not relevant, 2: somewhat relevant, 3: quite relevant, 4: highly relevant). This was repeated as many times as necessary until a consensus was reached on all items. The CVI value ranges from 0 to 1, where CVI values greater than 0.79 indicate that the item is relevant, those in the range of 0.70 and 0.79 indicate that the item needs updating, and those lower than 0.70 indicate that the item needs to be omitted [19,20]. Items with CVI values higher than 0.79 were kept in the final version of the scale. A total of 7 items were excluded because the condition of CVI** **>** **0.70 was not met. The reliability of the questionnaire was calculated with the Cronbach’s alpha internal consistency coefficient. The Cronbach’s alpha coefficient of the 26-item questionnaire was calculated as 0.827. All items on the questionnaire had high reliability. Cronbach's *α* values from 0.7 to 0.9 were considered acceptable, whereas values 0.6 to 0.7 were considered satisfactory.

Accordingly, the 26-item scale had a high level of reliability. In the scoring of the information form, the option “true” was worth 1 point, and the “false” and “I don't know” options were worth 0 points. The lowest and higher scores that could be obtained from the form were 0 and 26. Higher scores indicated higher knowledge levels.

#### 2.2.3. The form on the main traditional practices used when children have diarrhoea in Turkey

The form prepared by the researchers after the literature review consisted of 25 traditional methods known to be used by mothers in Turkey when children have diarrhoea [5,11,12,21,22]. The participants were asked to choose “yes” for the methods they knew and would use when their child had diarrhoea, “no” for the ones they knew but would not use, and “I have no information on this method” for the ones they did not have any knowledge about.

### 2.3. Data analysis

The data that were collected in the study were analysed using IBM SPSS v.23.0 (IBM Corp. Released 2015. IBM SPSS Statistics for Windows, Version 23.0. Armonk, NY: IBM Corp.). The normality of the distribution of the data was analysed using Shapiro–Wilk test. The results are presented as mean ± standard deviation or frequency and percentage. One-way analysis of variance (ANOVA) was used to compare the total DKS between education levels. Forward stepwise multiple linear regression analysis was used to determine the relationship between the total DKS and the other variables. The significance level was determined as *α*** **=** **0.05.

### 2.4. Ethical approval

In order to conduct the study, ethical approval was obtained from the Scientific Research and Publication Ethics Committee of Bursa Uludağ University (Date: August 26, 2020; No: 2020-07/04).

## 3. Results

The mean age of the mothers participating in the study was 32.37** **±** **4.63, their mean age of marriage was 25.03** **±** **4.00, and their mean age at first childbirth was 27.16** **±** **4.83. The mean number of children of the mothers was 1.59** **±** **0.71, and the mean number of their children under the age of 5 was 1.18** **±** **0.40. In the study, the mean age of the children under 5** **years of age was 25.94** **±** **18.31** **months. It was determined that 14.4% of the mothers were primary school graduates, 98.6% were married, 13% earned less than their expenses, 10.6% had extended families, 95.1% had health insurance, and 59.8% were working in a full-time job. The rate of the mothers whose children had diarrhoea at least once in their lifetime was 77.5%, and the rate of the mothers whose children had diarrhoea in the last month was 22.5%. Among the mothers, 35.9% stated that they breastfed their children until the age of 2. Table 1 presents the distribution of the participating mothers' socio-demographic characteristics.

**Table 1.** Distribution of the participants’ sociodemographic characteristics.

Variables	*n* = 577	
Age	32.37** **±** **4.63	
Age of marriage	25.03** **±** **4.00	
Age at first childbirth	27.16** **±** **4.83	
Total number of children	1.59** **±** **0.71	
Number of children under 5** **years old^a^	1.18** **±** **0.40	
Age of the child under 5** **years old (months)	25.94** **±** **18.31	
Mother’s education level (years)	14.97** **±** **5.16	
**Education level**		
Primary-Secondary School	83	14.4
High School	103	17.9
Bachelor’s Degree	265	45.9
Postgraduate Degree	126	21.8
**Marital status**		
Married	569	98.6
Single	8	1.4
**Family income**		
Income less than expenses	75	13.0
Income equal to expenses	328	56.8
Income more than expenses	174	30.2
**Family type**		
Extended family	61	10.6
Nuclear family	516	89.4
**Employment status**		
Full-time	345	59.8
Housewife	232	40.2
**Health insurance**		
Insured	549	95.1
Uninsured	28	4.9
**Diarrhoea at least once in the child's life**		
Yes	447	77.5
No	130	22.5
**Diarrhoea in the past month**		
Yes	124	21.5
No	453	78.5
**Breastfeeding duration of the child**		
No breastfeeding	9	1.6
Up to 1** **month	26	4.5
Up to 6** **months	75	13.0
Until 1** **year old	87	15.1
Until 2** **years old	207	35.9
More than 2** **years	40	6.9
Still breastfeeding	133	23.1

^a^Mothers with more than one child under the age of 5 were asked to answer the questions considering the child who experienced diarrhoea most.

Descriptive statistics are presented as mean ± standard deviation and frequency (%).

**Figure 1.**
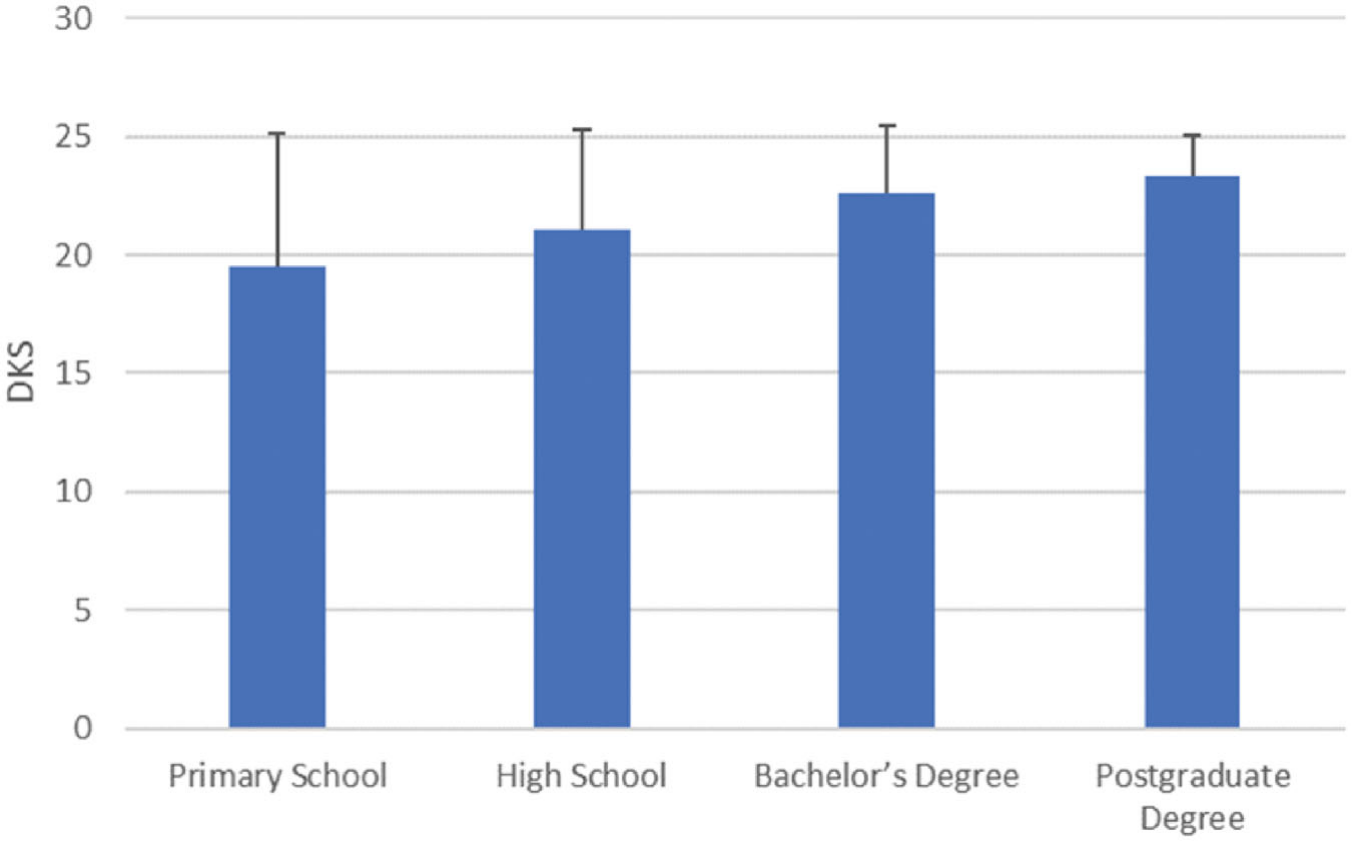
Diarrhoea knowledge score distribution according to education levels. *F* = 24.874; *p* < .001, DKS: Diarrhoea Knowledge Score.

In the study, the mean total diarrhoea knowledge score of the mothers was calculated as 22.01** **±** **3.72. When the diarrhoea knowledge score distribution was examined according to the education levels of the mothers, it was determined that the mean knowledge score of the mothers with a primary school education was 19.50** **±** **5.66, this score was 21.02** **±** **4.27 for the high school graduates, 22.56** **±** **2.88 for the university graduates, and 23.32** **±** **1.73 for the mothers with postgraduate education. The difference in the knowledge scores of the mothers based on their education levels was statistically significant (*p*** **<** **.001). While there was no statistically significant difference between the scores of the mothers with bachelor's degrees and those with postgraduate degrees, the scores of those with primary school or high school degrees were significantly different from those with other education levels. The distribution of the diarrhoea knowledge scores of the mothers according to their education levels is presented in Figure 1.

According to the multiple linear regression analysis, the variable that positively affected the diarrhoea knowledge score most was the status of having health insurance. Having health insurance increased the diarrhoea knowledge score by 2.86 points. Being a primary school graduate, on the other hand, reduced the diarrhoea knowledge score by 2.29 points, and it was the variable that negatively affected this score most. The multiple linear regression analysis results of the variables affecting DKS are presented in Table 2.

**Table 2.** multiple linear regression analysis results on the variables affecting DKS.

DKS	Non-standardized coefficients		Standardized coefficients	*T*	*p*
	Beta	Std. Error	Beta		
Constant	20.120	0.706	–	28.486	<.001
Family income (Income less than expenses)	−1.981	0.450	−0.179	−4.400	<.001
Health insurance (Insured)	2.862	0.689	0.165	4.151	<.001
Education level (Primary-Secondary School)	−2.229	0.445	−0.210	−5.014	<.001
Education level (High School)	−1.376	0.382	−0.142	−3.597	<.001

Model significance (*F*(4;572) = 29.263; *p*** **<** **.001), *R*^2^ = 0.170, Adjusted *R*^2^ = 0.164.

**Figure 2.**
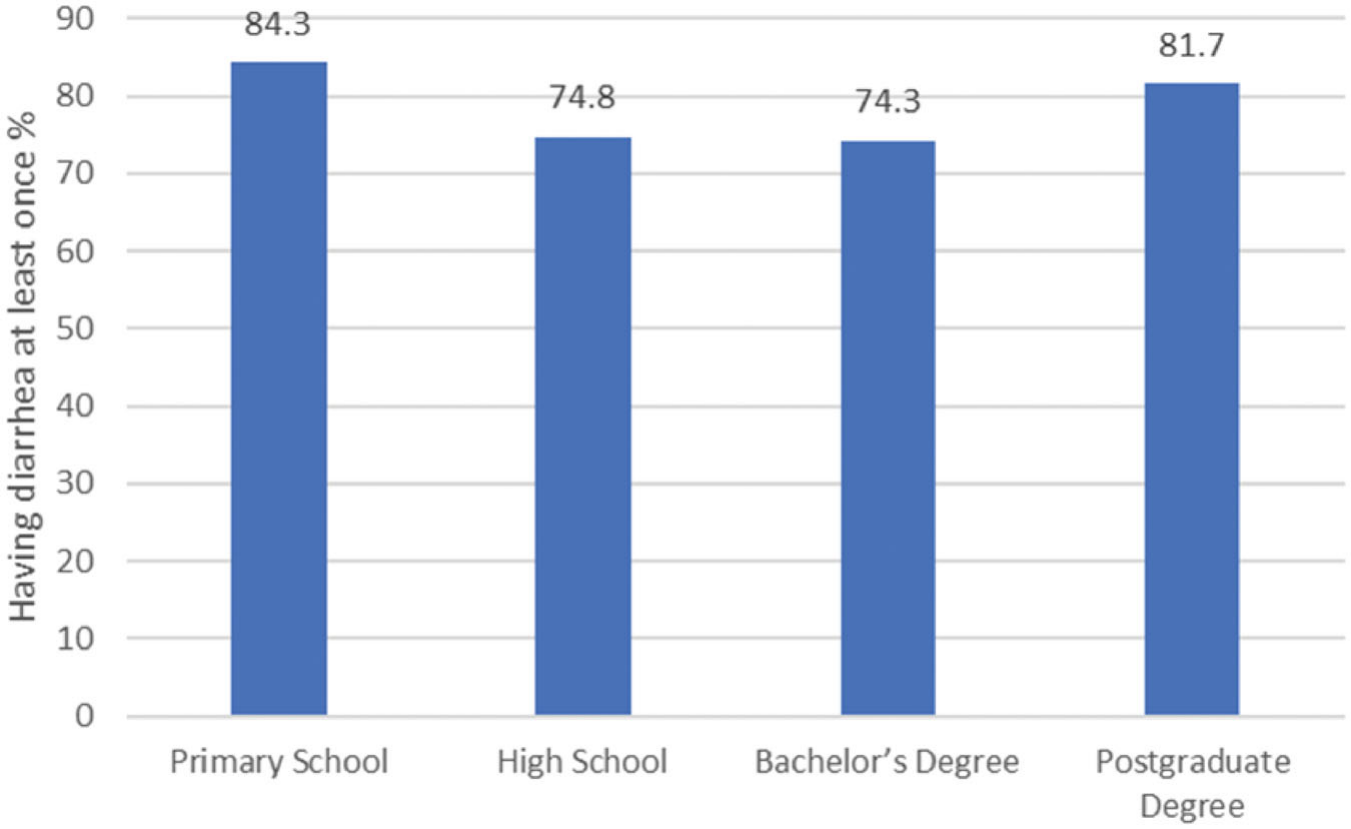
Distribution of the children having diarrhoea at least once, according to the education level of the mothers.

Regarding the responses of the mothers to the item “Diarrhoea at least once in the child's life”, it was seen that the children of 84.3% of the mothers with primary school education had diarrhoea at least once in their lifetime, while this rate was 74.8% for the mothers with high school degrees, 74.3% for the mothers with bachelor's degrees and 81.7% for the mothers with postgraduate degrees. The distribution of the rates of the children of the mothers having diarrhoea at least once in relation to the education levels of the mothers is presented in Figure 2.

The most prevalently preferred/would be preferred traditional practices by mothers in case of diarrhoea were determined as feeding the child banana (92.5%), feeding fat-free mashed potatoes (90.6%) and feeding rice porridge (79.0%). The main traditional practices that were or would be used by the mothers in this study when their children had diarrhoea are presented in Table 3.

**Table 3.** Distribution of the traditional practices preferred/would be preferred by the mothers when their children had diarrhoea (*n*** **=** **577).

No	The main traditional practices used when children have diarrhoea in Turkey	Yes *n* (%)
k1	Feeding the child banana^a^	534 (92.5)
k2	Feeding fat-free mashed potatoes^a^	523 (90.6)
k3	Feeding rice porridge^a^	456 (79.0)
k4	Giving the child boiled water to drink	336 (58.2)
k5	Feeding food with rice flour/custard^a^	293 (50.8)
k6	Feeding roasted chickpeas/chickpea powder^a^	217 (37.6)
k7	Applying warmth to the stomach	151 (26.2)
k8	Feeding a mixture of lemon and dry coffee^a^	122 (21.1)
k9	Giving a mixture of salt and sugar to drink^a^	110 (19.1)
k10	Giving the child sparkling mineral water^a^	92 (15.9)
k11	Praying for/asking someone to pray for the child	84 (14.6)
k12	Giving the child tea with lemon^a^	82 (14.2)
k13	Giving the child a mixture of soda and aspirin^a^	73 (12.7)
k14	Feeding a honey-ginger mixture^a^	61 (10.6)
k15	Feeding a mixture of yogurt and dry coffee^a^	60 (10.4)
k16	Giving the child sugar tea^a^	57 (9.9)
k17	Giving the child chamomile tea^a^	53 (9.2)
k18	Giving the child strong tea^a^	46 (8.0)
k19	Bathing the child in hot water	38 (6.6)
k20	Feeding dry tea leaves and cheese^a^	31 (5.4)
k21	Denying the child water	30 (5.2)
k22	Giving the child pickle juice^a^	25 (4.3)
k23	Giving the child a mixture of coconut, ginger and dry coffee to drink^a^	20 (3.5)
k24	Denying the child breastmilk	10 (1.7)
k25	Denying the child food	7 (1.2)

^a^Participants with children who had not switched to supplementary foods (from breastfeeding) were asked to give answers considering the time when they would switch. The letter “k” is descriptive code for each practice.

**Figure 3.**
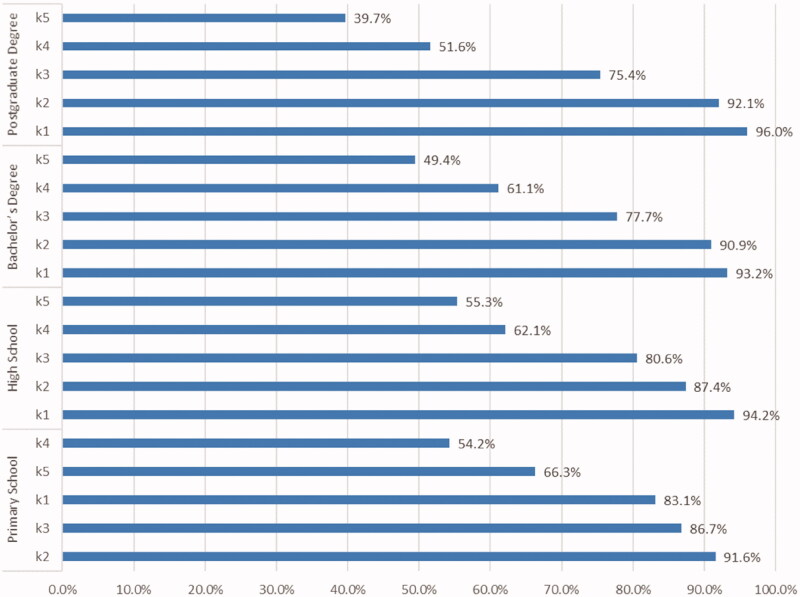
Top five traditional practices that the mothers preferred/would prefer most in the case of diarrhoea, according to their education levels. k1: Feeding the child banana, k2: Feeding fat-free mashed potatoes, k3: Feeding rice porridge, k4: Giving the child boiled water to drink, k5: Feeding food with rice flour/custard.

Regarding the distributions of the top five traditional practices that the mothers preferred/would prefer in the case of diarrhoea based on their education levels, the mothers with primary school degrees chose “feeding fat-free mashed potatoes” while the mothers with high school, bachelor's and postgraduate degrees chose “feeding the child banana”. Figure 3 presents the top five traditional practices that the mothers preferred/would prefer most in the case of diarrhoea in relation to their education levels.

## 4. Discussion

In this study, which was carried out to determine the diarrhoea-related knowledge levels and traditional practices of mothers with children under the age of 5, it was determined that the most significant variables affecting the diarrhoea knowledge scores of the mothers were the mother's education level, family income level and status of having health insurance. It was also determined that all mothers who participated in the study had a preferred traditional practice for when their children had diarrhoea.

Mothers are vital in the protection, continuation and improvement of the health of children under the age of 5, preventing diseases and caring for patients [9,10]. In the literature, it has been stated that the education level of mothers affects their behaviour of seeking health services for their children [9,14,15,23]. In this study, the mean total diarrhoea knowledge score of the mothers was determined as 22.01** **±** **3.72, and it was found that the mean knowledge score increased with education level (Figure 1). While similar results were reported in some studies [18,24–26], in others, the knowledge levels of mothers on diarrhoea were moderate [9,27]. In a previous study, only 36% of mothers were reported to have good diarrhoea management [28]. The differences between the knowledge levels of mothers in different studies might be related to geographical and cultural differences affecting access to information. In this study, it was determined that having only primary school education was the variable that affected the diarrhoea knowledge score most, and it reduced the score by 2.29 points (Table 2). When we look at some other studies, it may be seen that mothers' education levels positively affected their diarrhoea-related practices at home [28,29], their realisation of the severity of the disease [7,22,30], their behaviours of seeking medical help for diarrhoea [2,7,15,31] and the levels of their knowledge on the negative effects of diarrhoea [25,30]. These results may have been related to the easier access to information with increasing education levels, the mothers' knowledge about common health problems and their awareness about childcare. Unlike the findings of this study, some studies have not reported a relationship between education levels and diarrhoea knowledge/practices [6,32].

Although there was a positive significant relationship between the diarrhoea knowledge scores of the participants of this study and their education levels, there was no positive correlation in terms of the distribution of the statuses of the children of the mothers in terms of having diarrhoea at least once in the child's life and education levels (Figure 2). This result might have occurred in relation to the mothers' inability to convert their knowledge about diarrhoea into behaviour. In the study by Dodicho [33], although the rate of mothers with a good knowledge of diarrhoea treatment at home was high (67%), the ratio of bad diarrhoea management (52.8%) was high as well. Even though the results of this study seem to be similar to the results of Dodicho's [33] study, most people in Southern Ethiopia live in rural areas, and culture may affect behaviour.

In the literature, it has been reported that family income plays an important role in mothers' behaviours of seeking health services for their children [2,23,31]. In this study, family income was determined to be a factor affecting the diarrhoea knowledge scores of the mothers (Table 2). Similar results have been reported in different studies [7,28,31]. In the present study, it was also determined that the status of having health insurance was a factor affecting the diarrhoea knowledge scores of the mothers. The mothers who had health insurance had better knowledge about diarrhoea (Table 2). These results might have been related to the likelihood that families with health insurance have easier access to health services and obtain information about the health of their children from reliable sources.

In this study, it was found that all mothers in the sample knew about traditional practices, and they preferred/would prefer one of these practices if their children had diarrhoea. The top three traditional practices preferred/would be preferred by the participating mothers in the case of diarrhoea were determined as feeding the child banana (92.5%), feeding fat-free mashed potatoes (90.6%) and feeding rice porridge (79.0%) (Table 3). Studies conducted in Turkey and in developing countries have also reported that traditional practices are used for diarrhoea management. In these studies, the most frequently used methods have been stated as giving oral rehydration salts (ORS) [5,21,26,29,33–35], giving a salt-sugar mixture to drink [5,24,26,29,33], continuing to provide breast milk/to breastfeed [21,22,33,34], increasing fluid intake [29,34], giving rice water to drink [29,33–35], giving a mix of coffee and lemon to drink [5,21], giving a mix of soda and aspirin [5], feeding mashed potatoes [5,22], feeding food made with rice [5,21,22,26,27,34,35], feeding dough [27], giving fruit juice to drink [27], feeding dry and solid foods [5,21,24], feeding soups/liquid foods [24], feeding bananas [5,22,34,35], feeding yogurt [21,29], feeding porridge [35], continuing to feed normal homemade foods [34], feeding curd cheese [26], trying to cure diarrhoea using homemade remedies [14] and various herbs [25], withholding breast milk [27], limiting fluid intake [24,29], and limiting food intake [25], and some results of such studies have been in line with the results of this study. It seems that there are some similarities and differences among traditional practices preferred for diarrhoea management. In previous studies, it has been seen that there are different practices in the management of diarrhoea, and different variables such as the socio-demographic characteristics of mothers and the relationships of these variables to the symptoms of the disease are effective in the management of diarrhoea. It was seen in the literature review that studies have generally been carried out in countries with low socioeconomic levels. It is important to carry out studies on this topic in developed countries with high socio-cultural levels to make more accurate comparisons. The socio-cultural level of individuals is one of the effective factors in the management of diseases. However, as in the findings of this study, it should not be forgotten that there are traditional practices used also among mothers with high knowledge levels. It is important to raise the awareness of mothers about appropriate traditional practices to protect children's health and prevent diarrhoea-related complications.

## 5. Conclusion

In this study, the knowledge levels of the mothers on diarrhoea were determined to be high. The variables of education level, income level and health insurance status were significantly related to the mothers’ diarrhoea knowledge scores. It was determined that there were traditional practices that the mothers preferred/would prefer when their children had diarrhoea, and the most frequently preferred traditional practices were “feeding the child banana”, “feeding fat-free mashed potatoes” and “feeding rice porridge”. Although the participating mothers' levels of knowledge of diarrhoea were high in this study, the rate of their children having diarrhoea at least once in their life was also high. In order for mothers' knowledge about diarrhoea to be more effective and successful in practice, it is important to support learning processes on the affective and psychomotor levels. In addition to adequate and appropriate information, a good understanding of the aetiological factors and dynamics that cause diarrhoea is considered important in preventing diarrhoea-related complications. In this sense, questioning the factors affecting both mothers' knowledge levels about diarrhoea and their preferences to use traditional methods with qualitative studies may be useful in developing positive behaviours in diarrhoea management, determining positive and negative traditional practice preferences and adopting the positive ones among these.

## 6. Limitations

Illiterate mothers could not be included in the sample due to the online collection of data because of the conditions necessitated by the ongoing COVID-19 pandemic. Furthermore, the cross-sectional design of this study and the use of convenience sampling and online data collection were also limitations of the study.

## Data Availability

The data are available from the corresponding author upon reasonable request, provided that the necessary legal recourse is made.
